# Neuromuscular Strain Increases Symptom Intensity in Chronic Fatigue Syndrome

**DOI:** 10.1371/journal.pone.0159386

**Published:** 2016-07-18

**Authors:** Peter C. Rowe, Kevin R. Fontaine, Megan Lauver, Samantha E. Jasion, Colleen L. Marden, Malini Moni, Carol B. Thompson, Richard L. Violand

**Affiliations:** 1 Department of Pediatrics, Johns Hopkins University School of Medicine, Baltimore, Maryland, United States of America; 2 Department of Health Behavior, University of Alabama at Birmingham School of Public Health, Birmingham, Alabama, United States of America; 3 Department of Biostatistics, Johns Hopkins Bloomberg School of Public Health, Baltimore, Maryland, United States of America; 4 Rick Violand, PT LLC, Ellicott City, Maryland, United States of America; University of New South Wales, AUSTRALIA

## Abstract

Chronic fatigue syndrome (CFS) is a complex, multisystem disorder that can be disabling. CFS symptoms can be provoked by increased physical or cognitive activity, and by orthostatic stress. In preliminary work, we noted that CFS symptoms also could be provoked by application of longitudinal neural and soft tissue strain to the limbs and spine of affected individuals. In this study we measured the responses to a straight leg raise neuromuscular strain maneuver in individuals with CFS and healthy controls. We randomly assigned 60 individuals with CFS and 20 healthy controls to either a 15 minute period of passive supine straight leg raise (true neuromuscular strain) or a sham straight leg raise. The primary outcome measure was the symptom intensity difference between the scores during and 24 hours after the study maneuver compared to baseline. Fatigue, body pain, lightheadedness, concentration difficulties, and headache scores were measured individually on a 0–10 scale, and summed to create a composite symptom score. Compared to individuals with CFS in the sham strain group, those with CFS in the true strain group reported significantly increased body pain (P = 0.04) and concentration difficulties (P = 0.02) as well as increased composite symptom scores (all P = 0.03) during the maneuver. After 24 hours, the symptom intensity differences were significantly greater for the CFS true strain group for the individual symptom of lightheadedness (P = 0.001) and for the composite symptom score (P = 0.005). During and 24 hours after the exposure to the true strain maneuver, those with CFS had significantly higher individual and composite symptom intensity changes compared to the healthy controls. We conclude that a longitudinal strain applied to the nerves and soft tissues of the lower limb is capable of increasing symptom intensity in individuals with CFS for up to 24 hours. These findings support our preliminary observations that increased mechanical sensitivity may be a contributor to the provocation of symptoms in this disorder.

## Introduction

Chronic fatigue syndrome (CFS), often called myalgic encephalomyelitis/chronic fatigue syndrome (ME/CFS), is a complex, multisystem condition that is associated with a substantial impairment in pre-illness levels of activity and quality of life [1–3]. Individuals with CFS have increased symptoms during and after various physiologic challenges, such as physical exercise [4–6], orthostatic stress [7, 8], and cognitive tasks [9]. We recently reported that a different physiological challenge—neuromuscular strain—also has the potential to aggravate symptoms in those with CFS. In pilot work involving exposure to a 12 minute period of progressively increasing supine passive straight leg raise (SLR), two young adults with CFS developed a marked increase in fatigue, cognitive dysfunction, lightheadedness, and visual blurring [10]. These findings were extended in a larger study, in which 48 adolescents and young adults with CFS had significantly more areas of abnormal range of motion than healthy controls matched for sex and level of joint hypermobility. Moreover, in response to physical examination maneuvers that added an elongation strain to the nervous system and related soft tissues, CFS patients were more likely to develop abnormal symptomatic responses to the individual maneuvers and to the overall physical assessment [11]. Taken together, these observations are consistent with reduced compliance in the nervous system and its associated connective tissues in subjects with CFS, and with the hypothesis that increased mechanical sensitivity may be a contributor to the generation and exacerbation of CFS symptoms.

The current study was designed to further investigate this interaction between CFS symptoms and neuromuscular strain. We randomized individuals with CFS and healthy controls to either a true passive straight leg raise neuromuscular strain or a sham neuromuscular strain. We hypothesized that those with CFS exposed to the true strain would have a greater degree of symptom exacerbation than healthy controls exposed to the same strain, and that among those with CFS, individuals exposed to the true strain would report increased fatigue, body pain, lightheadedness, difficulty with concentration and headaches as well as a greater overall increase in CFS symptoms during the maneuver and 24 hour hours later compared to those undergoing the sham strain.

## Methods

### Study participants

Individuals with a physician diagnosis of CFS were eligible if they satisfied the 1994 International Chronic Fatigue Syndrome Study Group criteria for CFS [1] and were aged 16–55 years. Participants were asked to refrain from taking their morning doses of vasoactive drugs such as stimulants, modafanil, midodrine, beta-adrenergic antagonists, and salt tablets, but otherwise remained on their usual medications. Healthy controls were eligible if they were aged 16–55 years and reported good, very good, or excellent general health. Controls were also asked to skip morning doses of any of the vasoactive medications mentioned above.

Exclusion criteria included conditions expected to interfere with the passive SLR, such as recent lower limb or pelvic fractures, sprains, casting, any prior lower limb or pelvic surgery, known neuropathy, arthritis, cerebral palsy, developmental anomalies, pregnancy, or severe obesity. Healthy controls were excluded if they had self-reported CFS or fibromyalgia, postural tachycardia syndrome, neurally-mediated hypotension, recurrent syncope, or other health conditions that contribute to pain and fatigue.

Individuals with CFS were recruited from past CFS studies conducted by the investigators, as well as from the Johns Hopkins Pediatric Chronic Fatigue Clinic, media advertisements, targeted mailings, and CFS support groups. Controls were recruited from media advertisements and flyers circulated at the Johns Hopkins Hospital. Prospective participants were assessed during a phone screen. If they met eligibility criteria, they were invited to participate in the study, which was conducted during a half-day visit to the hospital. Written informed consent was obtained from all study participants 18 years of age or older. Those under 18 years provided verbal assent and written informed consent was granted by the parent. The study was approved by the Johns Hopkins University School of Medicine Institutional Review Board.

### Study design

**Fig 1:**
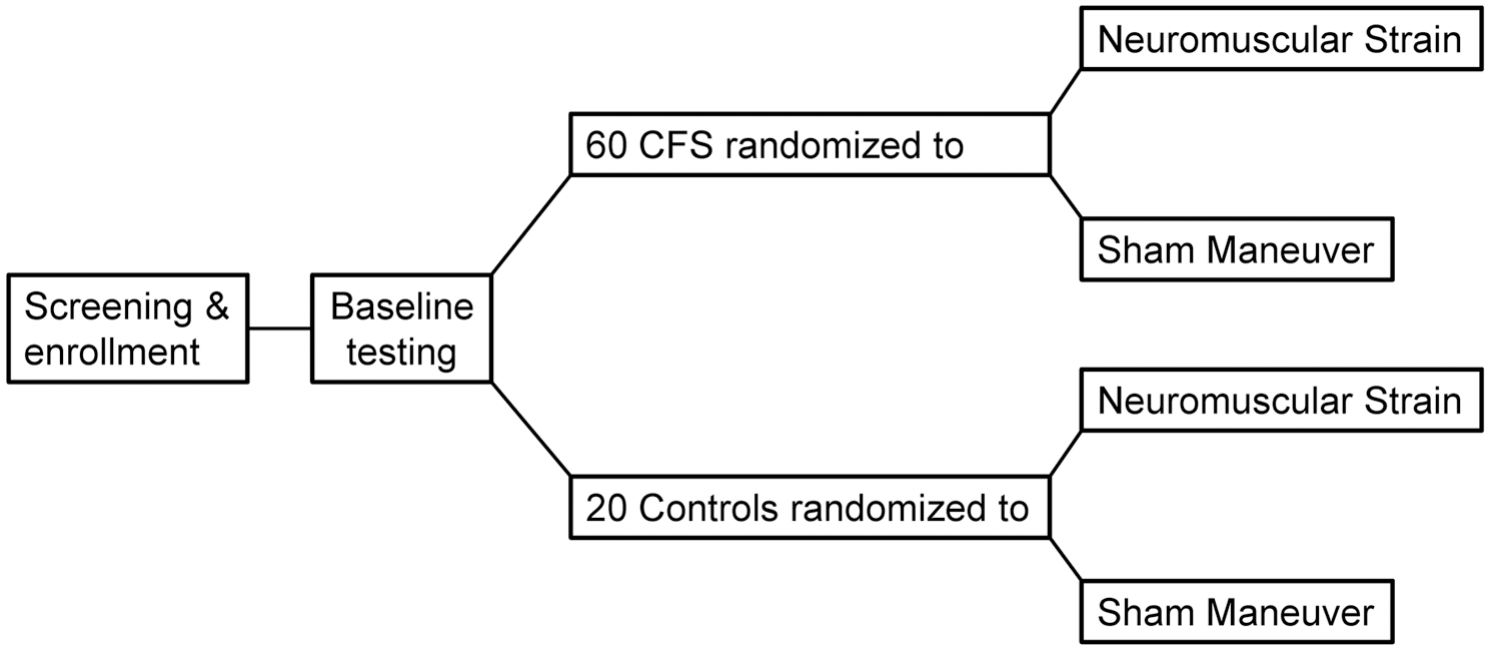
Study design.

Following the completion of a battery of baseline questionnaires and baseline examination measurements (described below), participants were positioned supine at rest for 15 minutes to allow heart rate (HR) and blood pressure (BP) to stabilize. They were then exposed to either a 15-minute true neuromuscular strain or a 15-minute sham test (Fig 1) as determined by the randomization code.

Heart rate, BP, and symptom intensity ratings were assessed every 5 minutes during and 5 minutes after completion of the strain or sham maneuver. Twenty-four hours after the study visit, participants were contacted by telephone and asked to rate their symptoms.

### Baseline questionnaires

Participants completed the following assessments of health-related quality of life before undergoing the strain or sham maneuvers:

1. Wellness score, a valid and reliable single-item measure that asks respondents to numerically rate their general sense of well-being over the past month, with 0 representing death and 100 representing as good as a person could feel [12, 13].
2. Multidimensional Fatigue Inventory (MFI), a 20-item scale that examines five dimensions of fatigue [14]. Higher scores indicate worse fatigue.
3. Symptom intensity, a subjective numerical score (on a 0–10 scale, with 0 indicating absence of the symptom and 10 indicating its greatest intensity) assigned by the respondent to symptoms of fatigue, body pain, lightheadedness, difficulty with concentration, and headaches.
4. Wood Mental Fatigue Inventory (WMFI), a subjective rating scale for nine mental fatigue symptoms. Higher scores indicate worse cognitive difficulty [15].
5. Center for Epidemiologic Studies-Depression Scale (CES-D), a 20-item questionnaire which measures depressive mood [16]. Higher scores indicate a greater burden of depressive symptoms.
6. Beck Anxiety Inventory (BAI), a 21-item scale with each item scored on a scale of 0–3. Scores of 0–21, 22–36, >36 indicate very low, moderate, and high anxiety, respectively [17].

### Baseline examinations

All participants underwent a tender point examination for the presence or absence of tenderness at the 18 sites specified in the 1990 American College of Rheumatology fibromyalgia classification criteria [18]. Testing for joint hypermobility was also performed using the 9-point Beighton scoring method [19].

### Strain and sham maneuvers

Participants were informed that the purpose of the study was to measure the way in which placing a strain on the nerves and muscles of the lower limb affected common CFS symptoms. Participants were randomized 1:1 within each of the two groups (CFS and healthy controls) to a true neuromuscular strain or a sham strain, each lasting 15 minutes. To ensure that range of motion was similar between strain and sham groups and to compare range of motion between those with CFS and healthy controls, all participants underwent measurement of passive SLR range of motion before the study procedure. During the SLR range of motion measurement, participants were instructed to report when they felt any ipsilateral lower limb stretch at all, even a slight one, and this was deemed the onset of stretch. The hip angle for the onset of stretch was recorded, as was the end-range angle. End-range was defined as the hip angle at which the participants indicated they did not want the limb raised further.

Because range of motion varies among individuals, we did not specify a pre-determined angle of SLR for the neuromuscular strain, as a given angle might create an insufficient strain for those with increased range of motion, and might be excessively uncomfortable for those with restricted range of motion. We therefore chose the mid-way point between the onset of stretch and end-range as the angle of the passive SLR for the true neuromuscular strain group.

For the 15 minute passive SLR strain maneuver, the research staff passively supported the non-dominant limb at the heel with the participant's hip flexed and knee extended at the hip angle mid-way between the onset of stretch and end range. For the 15 minute sham SLR maneuver, the research staff positioned the non-dominant limb in 5 degrees of hip flexion with rolled towels placed behind the knee and ankle to minimize the neural strain and the likelihood of associated symptom generation. Participants were instructed to not actively assist with muscle contraction to maintain the limb position. Extraneous limb movements were discouraged, and conversation between staff members and the participant was limited to essential instruction only.

Heart rate and BP were measured at baseline and once every 5 minutes with an automated sphygmomanometer for the duration of the test. At baseline, every 5 minutes during the maneuver, and 5 minutes afterwards, the research assistant asked participants to verbally report their symptom intensity scores at that moment on the 0–10 scale. The verbal response were chosen because completing written responses to the symptom intensity questions was impractical for participants when positioned supine, and would have superimposed a neuromuscular upper limb strain that would have interfered with measuring the response to SLR.

All participants were asked to refrain from moderate strenuous physical activity in the period between the end of the testing and their follow-up phone call 24 hours after the testing. At 24 hours, participants were asked to provide their current symptom intensity ratings using the 0–10 scale, as well as to complete two brief questionnaires, namely the Wellness Score and the WMFI.

### Sample size and randomization procedures

The main comparison of interest was the difference in symptom scores between individuals with CFS exposed to the true neuromuscular strain or to the sham maneuver. We considered a 10% higher symptom score (for any of the five CFS symptoms, namely fatigue, body pain, lightheadedness, difficulty with concentration, and headaches) during the study maneuver and 24 hours later to be clinically significant. A sample size of 30 per group for those with CFS had an 80% power to detect a 10% higher increase in symptoms in those exposed to the true SLR neuromuscular strain.

We chose a healthy control group to assess whether the impact of the study maneuvers on those with CFS differed from the impact on healthy individuals. Based on pilot work showing minimal impact on healthy individuals, we estimated that 20 healthy controls would answer this question.

Two separate randomization sequences were maintained, one for those with CFS and one for healthy controls. Within these categories, the randomization to strain or sham groups was conducted using a table of random numbers generated by one of the investigators (KRF). The randomization was not blocked. The assignment to strain or sham groups was kept in sequentially numbered opaque envelopes, which were opened by the research assistant after the participant had been enrolled.

### Statistical analysis

Baseline characteristics between individuals with CFS and healthy controls were compared with t-tests, or Mann-Whitney U tests depending on the data’s distribution. Categorical characteristics were compared using the Chi-square test or Fischer’s exact test depending on the distribution across categories. In addition to individual symptom intensity scores, we created a composite symptom score by summing the five individual symptom scores (maximum score = 50). To evaluate changes in individual symptom intensity scores from the baseline score in response to the neuromuscular strain maneuver, we calculated two change scores. One change score was based on the highest symptom intensity score reported during the strain or sham maneuver, and the other was based on the score at 24 hours after the procedure. In comparing changes in individual symptom intensity scores for fatigue, body pain, lightheadedness, difficulty with concentration, and headaches, we performed a two-factor (CFS vs healthy control and type of strain) regression analysis with an interaction term adjusting for their baseline values. The numbers of symptoms with at least 2-point increases in intensity were compared across the groups with the Chi-square test. The analyses were performed using STATA v12 (STATA Corp, College Station, TX) and SPSS v23 (IBM SPSS Statistics). Statistical significance was P<0.05. Marginal statistical significance was between 0.10 and 0.05.

## Results

### Participants

Eighty individuals (60 with CFS and 20 healthy controls) enrolled in the study and all completed the study protocol. Only 5 subjects in our study had been enrolled in the pediatric CFS clinic or had participated in prior pediatric CFS studies at Johns Hopkins. There was no difference between CFS and healthy control groups with regard to demographic variables (Table 1). As expected, on all measures of quality of life, individuals with CFS reported significantly worse function than healthy controls (P < 0.001). The mean (SD) angle of the mid-point between the onset of stretch and end-range for the SLR was 71 (14) degrees for healthy controls and 61 (17) for those with CFS (P = 0.02). For 88% of participants, the non-dominant leg was the left leg.

**Table 1:** Baseline characteristics of the study population.

Demographic features	Controls (N = 20)	CFS (N = 60)	P
	Mean (SD)^a^	Mean (SD)	
Female, N (%)	19 (95%)	51 (85%)	0.44
Age	37.5 (9.2)	36.9 (10.4)	0.82
College graduate, N (%)	12 (60%)	32 (53%)	0.80
Race, N (%)			0.86
White	15 (75%)	45 (75%)	
Black	3 (15%)	8 (13%)	
Asian	2 (10%)	5 (8%)	
Other	0 (0%)	2 (3%)	
BMI	25.5 (4.6)	26.7 (6.0)	0.42
Disabled, N (%)	0 (0%)	15 (25%)	0.02
Duration of CFS, years		5.6 (5.0)	
**HRQOL Measures**			
Wellness score	88 (12)	53 (16)	<0.001
Wood Mental Fatigue Inventory	3.1 (5.3)	19.1 (9.1)	<0.001
Multidimensional Fatigue Inventory			
General fatigue	9.1 (3.9)	17.4 (2.6)	<0.001
Physical fatigue	7.1 (3.2)	16.0 (3.6)	<0.001
Reduced activity	7.2 (3.4)	14.3 (4.2)	<0.001
Reduced motivation	7.6 (2.9)	12.2 (4.5)	<0.001
Mental fatigue	6.9 (3.3)	14.6 (3.6)	<0.001
CES-D	6.6 (6.7)	23.1 (11.9)	<0.001
Beck Anxiety Inventory	4.0 (3.9)	22.6 (11.1)	<0.001
**Examination characteristics**			
Beighton score, median (range)	0 (0–3)	1 (0–9)	0.11
Tender points at baseline	1.4 (1.7)	15.9 (3.3)	<0.001
Heart rate at baseline	66 (8)	72 (12)	0.06
Systolic BP at baseline (torr)	111 (12)	118 (15)	0.04
Diastolic BP at baseline (torr)	66 (9)	71 (9)	0.03

^a^ All values are mean (SD) unless otherwise indicated.

HRQOL, health-related quality of life; CES-D, Center for Epidemiologic Studies Depression Scale; BP, blood pressure.

The randomization showed similar results in demographic, quality of life, and examination variables between those in the strain or sham groups for both healthy controls and CFS participants (Table 2).

**Table 2:** Baseline characteristics after randomization.

Demographic features	CFS			Healthy Controls		
	Strain	Sham	P	Strain	Sham	P
	N = 32	N = 28		N = 9	N = 11	
	Mean (SD)^a^	Mean (SD)		Mean (SD)^a^	Mean (SD)	
Female, N (%)	26 (81%)	25 (89%)	0.48	8 (89%)	11(100%)	0.45
Age	34.9 (11.0)	39.2 (9.8)	0.11	39.1 (9.0)	36.2 (9.7)	0.50
College graduate, N (%)	19 (59%)	13 (46%)	0.42	7 (78%)	5 (46%)	0.20
Race, N (%)			0.58			0.24
White	23 (72%)	22 (79%)		8 (89%)	7 (64%)	
Black	4 (13%)	4 (14%)		0 (0%)	3 (27%)	
Asian	3 (9%)	2 (7%)		1 (11%)	1 (9%)	
Other	2 (6%)	0 (0%)		0 (0%)	0 (0%)	
BMI	27.2 (6.7)	26.1 (5.2)	0.51	24.9 (2.7)	26.0 (5.9)	0.60
Disabled, N (%)	6 (19%)	9 (32%)	0.38	N/A		
Duration of CFS, years	4.9 (4.4)	6.3 (5.5)	0.29	N/A		
**HRQOL Measures**						
Wellness score	54.2 (17.6)	52.4 (15.2)	0.69	85.4 (14.2)	90.2 (9.0)	0.38
Wood Mental Fatigue Inventory	19.2 (8.1)	19.0 (10.3)	0.92	5.1 ((7.1)	1.5 (2.4)	0.18
Multidimensional Fatigue Inventory						
General fatigue	17.4 (2.4)	17.3 (3.0)	0.87	9.7 (4.2)	8.6 (3.8)	0.57
Physical fatigue	15.8 (3.7)	16.2 (3.6)	0.67	6.9 (3.7)	7.3 (2.9)	0.80
Reduced activity	14.1 (4.6)	14.4 (3.9)	0.79	7.4 (4.3)	7.0 (2.8)	0.79
Reduced motivation	11.9 (4.8)	12.5 (4.2)	0.64	8.2 (2.7)	7.0 (3.0)	0.36
Mental fatigue	14.6 (3.5)	14.6 (3.9)	0.97	7.9 (4.2)	6.1 (2.3)	0.23
CES-D	21.7 (10.9)	24.7 (13.0)	0.35	7.7. (7.2)	5.6 (6.5)	0.52
Beck Anxiety Inventory	21.3 (11.8)	23.4 (10.4)	0.48	4.0 (2.3)	4.0 (5.0)	1.00
**Examination characteristics**						
Beighton score, median (range)	1.0 (0–9)	1.0 (0–9)	0.47	0.0 (0–2)	1.0 (0–3)	0.43
Tender points at baseline	15.8 (3.7)	16.0 (2.8)	0.80	2.0 (2.0)	1.0 (1.2)	0.15
Heart rate at baseline	71.9 (12.0)	71.3 (12.2)	0.84	66.7 (10.5)	65.7 (5.9)	0.80
Systolic BP at baseline (torr)	120.7(13.8)	115.9 (16.8)	0.22	109.4 (10.9)	111.5 (13.0)	0.70
Diastolic BP at baseline (torr)	71.1 (8.1)	70.5 (9.8)	0.80	65.9 (11.1)	65.3 (8.4)	0.89

^a^ All values are mean (SD) unless otherwise indicated.

HRQOL, health-related quality of life; CES-D, Center for Epidemiologic Studies Depression Scale; BP, blood pressure.

### Primary outcomes

The individual and composite symptom intensity scores at baseline, at the 5, 10, and 15 minute points during the strain maneuver, and at 5 minutes and 24 hours afterwards are shown in Table 3. Two factors limited the ability to identify a true change in symptoms using unadjusted statistical techniques. First, fatigue intensity scores were significantly higher at baseline for the CFS sham group than the CFS strain group (6.54 [1.67] vs. 5.38 [2.34]; P = 0.03). Second, among the 60 CFS participants, 17% had at least one symptom score of 9 or 10 at baseline, which thus limited their ability to report an increase in these symptoms due to the ceiling effect of the 0–10 scale. As a result, we used a regression analysis to examine the differences in symptom intensity change after adjusting for baseline values.

**Table 3:** Unadjusted comparisons of individual and composite symptom intensity scores before, during, and after the neuromuscular strain maneuver^a^.

	CFS			Healthy control	
Individual symptoms	StrainN = 32	ShamN = 28	P, CFS strain vs. CFS sham	StrainN = 9	ShamN = 11
** Fatigue**					
before	5.38 (2.34)	6.54 (1.67)	0.03	1.11 (1.76)	0.73 (1.49)
during, at 5 minutes	6.09 (2.22)	6.75 (1.80)	0.22	1.11 (2.32)	0.91 (1.70)
during, at 10 minutes	6.56 (2.26)	6.75 (1.84)	0.73	1.22 (2.39)	0.64 (1.57)
during, at 15 minutes	6.88 (2.67)	6.86 (2.10)	0.98	1.22 (2.39)	0.73 (1.68)
at 5 minutes post	6.77 (2.76)	6.96(2.08)	0.76	1.33 (2.50)	0.45 (1.51)
at 24 hours post	6.88 (2.25)	6.68 (1.83)	0.72	1.00 (1.66)	0.45 (1.51)
** Body pain**					
before	4.69 (2.47)	4.68 (2.86)	0.99	0.22 (.67)	0.36 (1.21)
during, at 5 minutes	5.25 (2.48)	4.79 (2.89)	0.51	0.11 (.33)	0.45 (1.51)
during, at 10 minutes	5.72 (2.85)	4.96 (3.10)	0.33	0.22 (.44)	0.45 (1.51)
during, at 15 minutes	6.22 (2.89)	5.18 (3.16)	0.19	0.22 (.44)	0.45 (1.51)
at 5 minutes post	5.58 (2.88)	5.11 (3.20)	0.55	0.11 (.33)	0.45 (1.51)
at 24 hours post	6.09 (2.76)	5.32 (2.98)	0.30	0.56 (.88)	0.91 (2.02)
** Lightheadedness**					
before	2.00 (2.50)	2.25 (2.65)	0.71	0 (0.0)	0.18 (0.60)
during, at 5 minutes	2.69 (2.67)	2.79 (2.73)	0.89	0 (0.0)	0.09 (0.30)
during, at 10 minutes	3.25 (2.66)	3.29 (3.05)	0.96	0 (0.0)	0 (0.0)
during, at 15 minutes	3.66 (3.00)	3.46 (3.11)	0.81	0 (0.0)	0 (0.0)
at 5 minutes post	3.63 (3.16)	3.48 (2.89)	0.84	0 (0.0)	0 (0.0)
at 24 hours post	3.53 (3.03)	1.96 (2.32)	0.03	0 (0.0)	0 (0.0)
** Difficulty with concentration**					
before	3.34 (2.57)	3.32 (3.01)	0.98	0.78 (2.33)	0.18 (0.60)
during, at 5 minutes	4.13 (2.76)	3.79 (3.10)	0.66	0.89 (2.32)	0.09 (0.30)
during, at 10 minutes	4.59 (2.88)	3.93 (3.10)	0.39	0.89 (2.32)	0.09 (0.30)
during, at 15 minutes	5.00 (2.63)	4.00 (2.96)	0.17	0.78 (2.33)	0.09 (0.30)
at 5 minutes post	4.41 (2.87)	4.18 (3.07)	0.77	0.78 (2.33)	0 (0.0)
at 24 hours post	4.75 (3.03)	4.07 (2.85)	0.38	0.67 (1.66)	0.09 (0.30)
** Headache**					
before	2.53 (2.51)	1.79 (2.57)	0.26	0 (0.0)	0.09 (0.30)
during, at 5 minutes	2.97 (2.69)	2.46 (3.18)	0.51	0 (0.0)	0 (0.0)
during, at 10 minutes	3.16 (2.89)	2.64 (3.01)	0.50	0 (0.0)	0 (0.0)
during, at 15 minutes	3.28 (2.93)	2.68 (3.02)	0.44	0 (0.0)	0 (0.0)
at 5 minutes post	3.09 (2.62)	2.86 (2.97)	0.74	0 (0.0)	0 (0.0)
at 24 hours post	3.31 (2.75)	2.75 (2.58)	0.42	0.67 (2.00)	0 (0.0)
**Composite symptom scores**^b^					
before	17.94 (9.46)	18.57 (8.11)	0.78	2.11 (3.72)	1.55 (2.77)
during, at 5 minutes	21.13 (9.89)	20.57 (9.02)	0.82	2.11 (4.51)	1.55 (3.05)
during, at 10 minutes	23.28 (10.46)	21.57 (9.40)	0.51	2.33 (4.50)	1.18 (3.06)
during, at 15 minutes	25.03 (10.71)	22.18 (9.80)	0.29	2.22 (4.55)	1.27 (3.13)
at 5 minutes post	23.47 (11.14)	22.57 (9.73)	0.74	2.22 (4.63)	0.91 (3.02)
at 24 hours post	24.56 (10.62)	20.79 (7.42)	0.11	2.89 (3.52)	1.45 (3.50)

^a^ Values are mean (SD).The comparisons between healthy control strain and healthy control sham groups are provided to display general trends, but the sample size was insufficient for statistical comparisons.

^b^ The composite symptom score was created by summing each of the individual scores for fatigue, body pain, lightheadedness, difficulty with concentration, and headache (maximum score = 50).

### Changes in individual symptom intensity scores during and 24 hours after the maneuver

CFS Strain vs. Control Strain: The mean differences between groups for changes in symptom intensity (the highest symptom score during the neuromuscular strain maneuver or at 24 hours minus the baseline score) are shown in Table 4. Compared to healthy controls exposed to the 15 minute SLR, CFS participants exposed to the same neuromuscular strain maneuver had a significantly greater mean change in intensity of all symptoms, adjusted for baseline values, during the maneuver (Table 4). For example, the mean (95% confidence interval [CI]) change in fatigue intensity was 2.78 (0.35, 5.20) points higher in CFS participants than in healthy controls, and the mean (95% CI) change in body pain severity was 2.05 (0.87, 3.23) points higher in CFS participants than in controls. Similarly, at 24 hours post-maneuver, the CFS strain group had significantly greater change in symptom intensity compared to healthy controls exposed to the strain maneuver for all symptoms except headaches.

**Table 4:** Mean difference and 95% confidence intervals (CI) between groups for changes in symptom intensity scores during and 24 hours after the maneuver, adjusted for baseline values.

**CFS strain minus healthy control strain**				
	**During 15 minute strain**		**24 hours after strain**	
*Symptoms*	**Mean difference (95% CI)**	**P-value**	**Mean difference (95% CI)**	**P-value**
Fatigue	2.78 (0.35, 5.20)	0.03	3.42 (1.62, 5.22)	<0.001
Body pain	2.05 (0.87, 3.23)	0.001	1.87 (0.49, 3.25)	<0.01
Lightheadedness	2.36 (1.37, 3.35)	<0.001	2.05 (1.11, 2.98)	<0.001
Difficulty with concentration	2.57 (1.69, 3.44)	<0.001	2.07 (1.19, 2.95)	<0.001
Headaches	0.90 (0.20, 1.59)	0.01	0.88 (-0.65, 2.41)	0.26
Composite symptom score	9.46 (4.81, 14.12)	<0.001	8.71 (5.07, 12.35)	<0.001
**CFS strain minus CFS sham**				
	**During 15 minute maneuver**		**24 hours after maneuver**	
*Symptoms*	**Mean difference (95% CI)**	**P-value**	**Mean difference (95% CI)**	**P-value**
Fatigue	0.72 (-0.01, 1.45)	0.05	0.86 (-0.05, 1.78)	0.07
Body pain	0.94 (0.07, 1.81)	0.04	0.76 (-0.21, 1.74)	0.12
Lightheadedness	0.63 (-0.48, 1.74)	0.26	1.75 (0.78, 2.72)	0.001
Difficulty with concentration	1.13 (0.17, 2.08)	0.02	0.66 (-0.35, 1.67)	0.20
Headaches	-0.23 (-1.1, 0.64)	0.60	0.04 (-1.03, 1.12)	0.94
Composite symptom score	3.52 (0.41, 6.62)	0.03	4.30 (1.32, 7.27)	0.005

CFS Strain vs. CFS Sham groups: As shown in Table 4, during the maneuver, the mean (95% CI) difference between baseline and peak symptom intensity was 0.94 (0.07, 1.81) points greater in the CFS strain group than in the CFS sham group for body pain, and 1.13 (0.17, 2.08) points higher in the CFS strain group for difficulty with concentration. The mean difference in fatigue severity was 0.72 (-0.01, 1.45) points higher in the strain group (marginally significant). Changes in the intensity of lightheadedness and headache did not differ between groups during the 15 minute maneuver.

Despite no difference in change for lightheadedness during the maneuver, at 24 hours post-maneuver, those in the CFS strain group had mean (95% CI) scores 1.75 (0.78, 2.72) points higher for lightheadedness. No other changes in individual symptom intensity scores differed between groups at 24 hours post-maneuver.

### Changes in composite symptom intensity scores during and 24 hours after the maneuver

CFS Strain vs. Healthy Control Strain: As shown in Table 4, the mean (95% CI) difference in change of the composite symptom score during the maneuver was 9.46 (4.81, 14.12) points higher in CFS participants than in the healthy control group (P<0.001). The mean difference in composite symptom scores at 24 hours compared to baseline was also higher in individuals with CFS than healthy controls exposed to the true strain maneuver (P<0.001).

CFS Strain vs. CFS Sham groups: CFS strain participants had a significantly greater mean (95% CI) difference of 3.52 (0.41, 6.62) points in the composite symptom score during the maneuver than CFS participants exposed to the sham maneuver (Table 4). The mean (95% CI) difference in composite symptom score was 4.30 (1.32, 7.27) points higher in the CFS strain than the CFS sham group at the 24 hour point.

### Proportions with changes in symptom intensity scores of ≥ 2 points during and 24 hours after the maneuver

**Fig 2:**
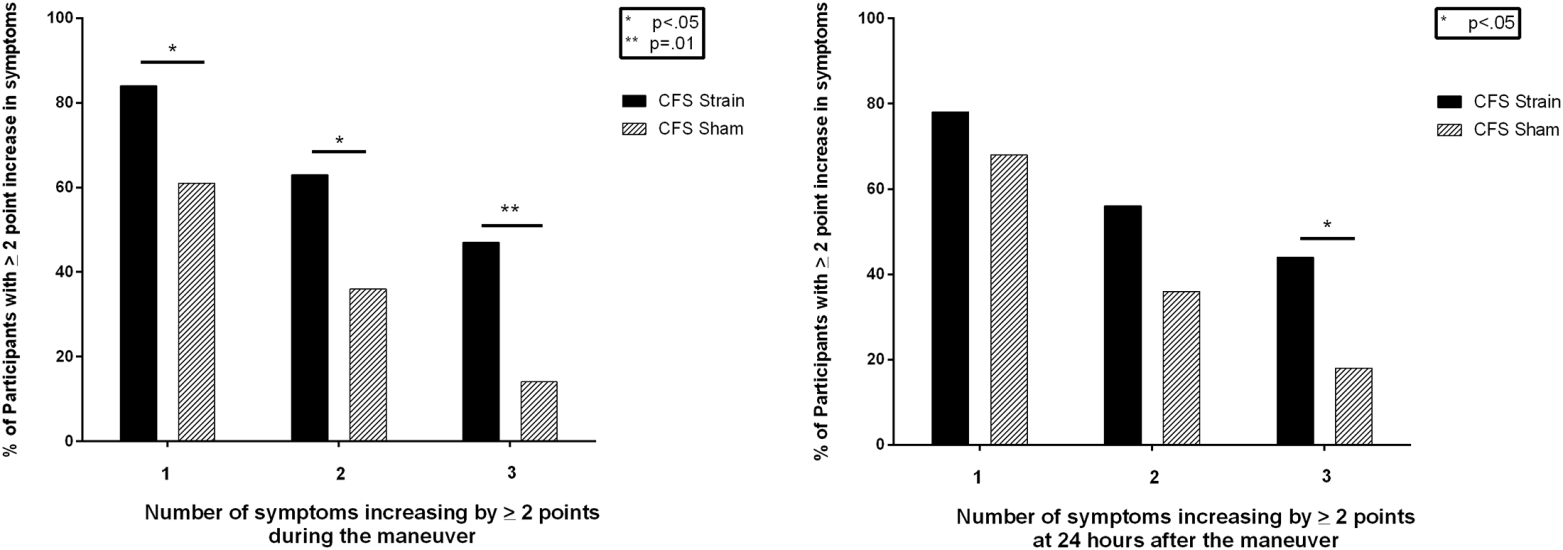
Proportion of CFS participants reporting a ≥ 2 point increase in symptom intensity for any 1, 2, or 3 symptoms (a) during the straight leg raise or sham neuromuscular strain or (b) 24 hours post-maneuver.

The high baseline scores of 9 or 10 for at least one symptom in 17% of those with CFS created a potential to underestimate the magnitude of change in symptoms in response to the maneuver due to a ceiling effect. To address this, we also performed a post-hoc analysis of the proportion of subjects reporting at least a 2-point increase in at least 1 symptom, at least 2 symptoms, or at least 3 symptoms during and 24 hours after the maneuver. As shown in Table 5 for all groups, and as illustrated in Fig 2 for the CFS participants, a significantly higher proportion of the CFS strain group than the CFS sham group had at least a 2-point increase in symptom intensity for at least 1 symptom (84% vs. 61%; P = 0.04), at least 2 symptoms (63% vs. 36%; P = 0.04), or at least 3 symptoms (47% vs 14%; P = 0.01). The difference between CFS strain and sham groups was significant at 24 hours post-test for a change of at least 2 points in at least 3 symptoms (44% vs. 18%; P = 0.03).

**Table 5:** Group comparisons of increases of at least 2 points in symptom intensity during and 24 hours after the maneuver.

	CFS		Healthy Control		P	
	Strain N = 32	Sham N = 28	Strain N = 9	Sham N = 11	CFS Strain vs. CFS Sham	CFS Strain vs. Healthy Control Strain
**During Maneuver**						
≥ 2 point increase in at least 1 symptom	84%	61%	22%	18%	0.04	0.001
≥ 2 point increase in at least 2 symptoms	63%	36%	0%	0%	0.04	0.001
≥ 2 point increase in at least 3 symptoms	47%	14%	0%	0%	0.01	0.02
**24 hours after maneuver**						
≥ 2 point increase in at least 1 symptom	78%	68%	44%	9%	0.37	0.09
≥ 2 point increase in at least 2 symptoms	56%	36%	11%	0%	0.11	0.02
≥ 2 point increase in at least 3 symptoms	44%	18%	0%	0%	0.03	0.02

The mean (SD) wellness score did not differ between CFS strain and sham groups at 24 hours after the study maneuver [43(18) vs. 48(22); P = 0.40], nor did the Wood Mental Fatigue Inventory score [17(9) vs. 15(8); P = 0.25].

### Secondary outcomes

#### Differences in heart rate and blood pressure

The mean (SD) systolic BP was similar between CFS strain and sham groups at baseline [121(14) vs. 116(17) torr; P = 0.22], as were the diastolic BP [71(8) vs. 71(10) torr; P = 0.80) and the HR [72(12) vs. 71(12) beats per minute; P = 0.84]. There were no differences between groups after 15 minutes of the study maneuver in these parameters (all P >0.10).

## Discussion

The main finding of this study is that a sustained longitudinal strain applied to the neural and soft tissues of the lower limb was associated with an increased intensity of cardinal CFS symptoms during the maneuver and for up to 24 hours afterwards. Those with CFS exposed to a passive SLR strain for 15 minutes had a significantly greater exacerbation in all symptoms compared to healthy controls exposed to the same strain, and a greater exacerbation of symptoms than those with CFS exposed to the sham strain. Among those with CFS, the difference between the SLR strain and sham groups was evident when comparing the individual evoked symptoms of body pain and impaired concentration for those groups and the combined symptom score. At 24 hours after the study maneuver, CFS subjects in the strain group had significantly more lightheadedness and a higher combined symptom score than those in the CFS sham group. These differences in symptom intensity scores were relatively modest. Changes in symptom intensity were more obvious when increases in symptom scores of at least 2 points were assessed. Compared to those in the CFS sham group, a significantly higher proportion of CFS subjects in the strain group reported increases of at least 2 points in at least 1, 2, or 3 symptoms during the study maneuver (47% vs. 14% for a change of at least 2 points in at least 3 symptoms; P = 0.01), and a higher proportion reported at least a 2-point increase in at least 3 symptoms at 24 hours (44% vs. 18%; P = 0.03).

Of interest, at 24-hours post-test, healthy controls in the strain group reported more symptom changes of at least 2 points in any symptom (44% vs 9%), a marginally significant difference. While the study was not designed to perform statistical comparisons between the two healthy control groups, the observed symptom changes suggest that the passive SLR strain maneuver might be a broad physiologic challenge for all, but like exercise, orthostatic stress, and cognitive stress, experienced at a much more profound level for those with CFS.

These findings complement earlier pilot data describing a marked change in fatigue, mental fogginess, and lightheadedness during a progressive increase in SLR angle over 12 minutes [10]. Our results also extend findings from an examination of limb and spine range of motion among 48 adolescents and young adults with CFS and 48 healthy controls matched by sex and the degree of joint hypermobility. In that investigation, we assessed range of motion in response to commonly-used physical examination maneuvers that add an elongation strain to the nervous system and related soft tissues, including a passive SLR of only several seconds duration. Of the 11 limb and spine regions examined, individuals with CFS had a higher median number of areas of abnormal range of motion (5 vs. 2, P< 0.001), and were more likely than healthy controls to develop abnormal symptomatic responses to range of motion testing [11].

### Biomechanical and physiological considerations with SLR

While the results of the current study confirm that individuals with CFS have an increased sensitivity to SLR compared to healthy individuals, the mechanisms for transduction of that neuromuscular strain into increased symptoms are unknown. The structural and biochemical responses of the nervous system to physical stress and elongation strain are relevant to this discussion. In the past several decades, work by Sunderland, Brieg, and others has investigated the interaction between nerve mechanics and nerve function [20–25]. The nervous system’s normal adaptation to the range of limb and trunk movement in daily life involves accommodative changes in length and nerve sliding within fascial beds. For example, the contents of the spinal canal (including neuromeningeal and vascular tissues) must elongate to adjust to as much as a 5–9 cm length change of the spinal canal as one moves in standing from a position of full spinal backward bend to full forward bend [20, 26]. The cord, meninges, and supporting vasculature must undergo changes in length to accommodate this canal lengthening without altering conductivity or other neural functioning.

It has been well established in cadaver studies that passive SLR exerts caudal traction on the lower limb peripheral nerves, dorsal root ganglia, lumbosacral nerve roots, meninges, and the sympathetic chain [20, 27–29]. The densely innervated thoracolumbar fascia would be expected to be subjected to the same forces [30]. Evidence from human cadaver specimens and from experiments in animals suggests that SLR also imparts an elongation strain to the entire length of the spinal cord [28]. More recently, caudal displacement of lumbosacral nerve roots and the spinal cord has been confirmed in living subjects. Using MRI techniques, Rade and colleagues identified a mean 3.5 mm caudal displacement of the conus medullaris during a 60° passive SLR [31]. Of interest, 60° of passive SLR is the same mean angular displacement recorded among the CFS participants in our study. Thus the neural and soft tissue strain in this maneuver could have exerted a similar caudal strain on the cord and its coverings in our participants.

Two further physiologic consequences of tension applied to the spinal cord during SLR can be inferred. First, increased mechanical strain within the spinal cord—which can be caused by acquired segmental, positional dysfunctions in the spine [32], or by tethering or deformative stresses affecting the spinal cord, dura, and sympathetic chain [20, 33, 34]—could cause a longitudinal tensioning of spinal blood vessels, the result of which is narrowing of lumen diameter [20]. In animal experiments, Dolan and colleagues demonstrated that application of a distraction strain to the spinal cord results in cord ischemia, with progressive impairment of spinal cord blood flow as the distraction strain increases [35]. Yamada and colleagues used reflection spectrophotometry to measure the availability of oxygen to neuronal mitochondria in an animal model of tethered cord syndrome. With increasing strain, there was evidence of increased reduction of cytochrome *a*, *a*_*3*_, reflecting mitochondrial dysfunction [36]. Kobayashi and colleagues demonstrated that intra-radicular blood flow is reduced by a mean of 71% compared to baseline during intra-operative SLR in those with nerve root compression due to bulging discs [37]. While the SLR imparted in our study would not have been expected to be severe enough to result in the ischemic change seen with distraction or tethered cord, or with the reduced nerve root blood flow seen with severe degenerative disc disease, these observations establish the potential for passive SLR strain to contribute to at least transient changes in endothelial shear stress and to transient reduction in blood flow to the nerve root and potentially to spinal cord neurons.

Second, tension from the SLR is transmitted from the epineurium of the sciatic nerve to the dural sleeves of the associated nerve roots and to the dural tube within the spinal canal [22, 26]. Mast cells are distributed throughout the nervous system, including in the dura. Mast cells are also known to degranulate in response to stretch [38, 39]. The release of histamine, prostaglandins, and other biologically active substances in mast cells would have the potential to cause both acute and delayed symptoms in response to SLR. Mast cell activation has been hypothesized to be a pathophysiologic factor in CFS [40], as well as in the pathogenesis of symptoms in subsets of patients with postural tachycardia syndrome [41] and joint hypermobility syndromes [42], two co-morbid conditions with an increased prevalence in CFS [43].

### Speculations about the pathophysiology of increased symptoms in response to SLR in CFS

In the context of these physiologic correlates of SLR, why would this maneuver be associated with only minimal perturbation in healthy individuals yet cause a significant exacerbation of symptoms in those with CFS? Current theories of the pathogenesis of CFS symptoms implicate heightened sensitivity of the nervous system to physical, orthostatic, and cognitive stressors [44]. Among the hypothesized mechanisms for nervous system sensitivity are (a) a persistent and abnormal stress response to a variety of precipitating and perpetuating conditions [45], (b) reductions in cerebral blood flow [46, 47], a downstream effect of which is an increase in anaerobic metabolism, supported by observations that ventricular lactate levels are higher in CFS patients than in either healthy or depressed controls [48], and (c) neuroinflammation in response to infection, auto-immune inflammation, or other factors [49]. In a small controlled study, Nakatomi and colleagues used PET scanning to identify increased binding of a ligand for an 18 kDa translocator protein (TPSO) that is expressed by activated microglia. Individuals with CFS had significantly higher binding than healthy controls in the midbrain, pons, and thalamus (all P<0.01); binding was closely correlated to the reports of fatigue severity, cognitive impairment, pain, and measures of depression [49]. While mechanisms for microglial activation were not investigated in the Nakatomi study, several of the proposed mechanisms for CFS symptoms would be consistent with changes in microglial activity, including reduced cerebral blood flow, sympathetic activation, mast cell activation, or sustained immunological responses to an initiating infection.

In light of these observations, we speculate that the increase in CFS symptoms after the imposition of an otherwise mild mechanical strain like SLR could be consistent with the presence of a non-compliant or mechanically sensitized nervous system. The same strain is well tolerated by those with a non-sensitized nervous system. The development of symptoms such as difficulty concentrating and body pain within 15 minutes of initiating the neuromuscular strain is consistent with SLR provoking an acute physiologic response, likely affecting autonomic tone or cerebral blood flow. The persistence or emergence of increased symptoms over the 24 hours following the neuromuscular strain is analogous to the gene expression changes reported in the 24–48 hours after exercise in CFS [5].

### Study limitations

This study provides an initial description of the response of those with CFS to SLR. It had several limitations. We are confident that the CFS participants in the SLR and sham strain groups had similar demographic characteristics, illness duration and severity, and health-related quality of life. This suggests that the randomization was successful, and that the results reflect differences in response to the study maneuver rather than differences in the severity of illness. It remains to be seen whether the results we report would be similar in other groups with CFS, in those with different durations of illness, or for individuals with different levels of physical function.

Similarly, fibromyalgia is a condition with a substantial overlap with CFS, estimated to affect 30–70% of adults with CFS [50, 51]. Some of our study population met criteria for fibromyalgia, but we made no attempt to differentiate this subset. Because fibromyalgia tender point scores did not differ between the CFS strain and sham groups at baseline, it is unlikely that an unbalanced assignment of those with fibromyalgia to either experimental group occurred. In future studies, the independent impact of fibromyalgia could be evaluated by including groups satisfying the CFS criteria only, fibromyalgia without CFS, or both conditions.

The study was randomized, but blinding subjects to the degree of SLR was not possible. The potential for biased reporting of symptom scores among those in the true strain group cannot be excluded.

We did not measure symptom changes beyond 24 hours, so we cannot make definitive comments about the duration of post-maneuver symptom exacerbations. Nonetheless, our results suggest that neuromuscular strain is another mechanism for the generation of post-exertional worsening of symptoms, regarded as a defining feature of CFS [2]. We also did not measure changes in biomarkers at 24 or 48 hours, but these would be important to include in future studies.

The impact of the neuromuscular strain may have been underestimated because some individuals had symptom intensity scores in the upper part of the 0–10 measurement range at baseline. Without an objective measure of fatigue and pain, we relied on verbal self-report of symptoms as the primary outcome. For those with a high baseline score of 9 or 10, it would have been impossible to measure whether symptoms became substantially worse following the SLR maneuver. In future studies, potential ways to address this problem would be to exclude subjects with the highest baseline symptom ratings, to ask participants to report the degree of improvement or exacerbation of symptoms on a Likert-scale, or to include a patient and clinician global clinical impression of change score at the end of the maneuver that would supplement the symptom intensity scores. Validated measures of symptom change such as the Fatigue and Energy Scale [52] have been published since our study was designed, and have the potential to provide an improved method of measuring change following provocation maneuvers.

In a study with randomized assignment to the physiological strain, any measured differences between the CFS strain and sham groups were likely to be related to the study maneuver. The randomization would have reduced the likelihood of marked variability in the degree of orthostatic stress, physical exercise, or cognitive stress in the 24 hours after the study visit. However, in the absence of an activity measure, we cannot exclude some influence of these factors on the 24 hour symptom scores.

Individuals with CFS can have impaired range of motion and mechanosensitivity in regions other than the lower limb [11] in which case the SLR maneuver might be insufficient to provoke symptoms. For this study, a simple and straightforward neuromuscular strain familiar to most clinicians was selected as a means of introducing the concept of mechanical strain. Future studies of the phenomenon we have observed could add different sites of neuromuscular strain, or add structural differentiation maneuvers that can better distinguish neural from myofascial sources of symptoms [53, 54]. Methods to refine the maneuver and enhance tissue specificity could include the addition of cervical flexion or ankle dorsiflexion while the limb is maintained in the SLR position [54]. A report of symptom increase or decrease with the addition of joint movement at locations beyond the points of attachment of the challenged muscles would implicate nerve rather than muscle involvement.

### Implications and future directions

Our findings have practical implications for the understanding of why exercise and the activities of daily life might be capable of provoking CFS symptoms. If a simple and relatively brief passive SLR strain can provoke symptoms, then prolonged or excessive strain beyond the usual range of motion in daily life might be followed by a similar exacerbation. A common position that approximates the hip angle of a SLR maneuver is long sitting, as can occur while reclining in bed with pillows to support the spine or when sitting with the right leg extended and arms outstretched while driving. These positions each would be expected to add mechanical tension to the nervous system. Such tension could be increased by ankle dorsiflexion or plantar flexion. Further, some sleep or reclining postures could increase CFS symptoms despite appearing to be positions of rest and recovery. Similarly, in a person with limited, but symptom-free SLR who walks or runs with long strides, repetitive elongation strains of the limbs or spine may readily induce CFS symptoms. Standing in stocking feet or in shoes with flat soles likewise increases mechanical tension in the nerve tissues. The presence of such a nuanced but profound means of symptom generation has practical implications for the care of affected patients. Those with more marked restrictions in range of movement, or greater symptomatic responses to longitudinal strain to the nerves and soft tissues, would be expected to have a greater difficulty tolerating exercise. It is reasonable to hypothesize that treating these areas of movement restriction—before advancing to more exertional activities—might improve the ability of CFS patients to tolerate graded exercise. Consistent with this hypothesis, in adults with a related condition, fibromyalgia, a randomized trial of neural mobilization treatments has shown improvements in pain, fatigue, and adverse neural tension [55]. Further studies are warranted to better understand the prevalence, risk factors, and impact of neuromuscular strain in CFS, and the optimal methods to restore more normal function to those with the illness.

## Supporting Information

S1 File SPSS study data file. (SAV)
